# First overall report of *Leptospira* infections in wild boars in Poland

**DOI:** 10.1186/s13028-016-0186-7

**Published:** 2016-01-12

**Authors:** Jacek Żmudzki, Artur Jabłoński, Agnieszka Nowak, Sylwia Zębek, Zbigniew Arent, Łukasz Bocian, Zygmunt Pejsak

**Affiliations:** 1 Swine Diseases Department, National Veterinary Research Institute, Partyzantow 57, 24-100 Pulawy, Poland; 2 Epidemiology and Risk Assessment Department, National Veterinary Research Institute, Partyzantow 57, 24-100 Pulawy, Poland; 3 OIE Leptospira Reference Laboratory, Veterinary Sciences Division, AFBI, Stoney Road, Stormont, Belfast, Northern Ireland BT43SD UK

**Keywords:** Leptospirosis, Wild boar, Serology, Prevalence, Zoonosis

## Abstract

**Background:**

Recently an increase in the population of wild boars (*Sus scrofa*) in Europe has been observed. This is important from a zoonotic perspective because it influences directly on the spread of many diseases. For the first time, an extensive survey on the prevalence of *Leptospira* infections in Polish wild boars was performed. During the hunting season 2012–2014, 3621 blood samples from wild boars were collected. The animals originated from different geographical areas across Poland. Serum samples were tested by a microscopic agglutination test (MAT) for the presence of specific antibodies to the following *Leptospira* serovars: Icterohaemorrhagiae, Grippotyphosa, Sejroe, Tarassovi, Pomona, Canicola, Bratislava, Autumnalis, Hardjo and Ballum.

**Results:**

Antibody titers to all *Leptospira* serovars except serovar Ballum were found in 377 serum samples (10.4 %). The highest number of seropositive wild boars was found in the south-eastern part of Poland and in highly urbanized areas such as Silesia and Łódź.

**Conclusions:**

The relatively high prevalence of *Leptospira* infections in wild boars may constitute a threat to hunters and people having contact with forest lakes or marshlands. The results also indicate that an increasing population of wild boar living close to borders of cities may create additional risk for inhabitants in large urban areas.

## Findings

Leptospirosis is a widely occurring zoonotic disease caused by pathogenic serovars of the genus *Leptospira*. Wild animals are considered important reservoirs of leptospirosis in humans and livestock. Among the wide range of game species, the wild boar (*Sus scrofa*) has been identified as an emerging problem in various European countries [1–6]. In the last decade favorable environmental conditions such as mild winters, regular feeding of animals by forest guards, large amounts of natural food available, and changes in agriculture related to intensive cultivation of corn have led to a significant increase in European population of wild boars [7].

Increasing populations of wild boars near suburban areas not only cause a direct threat to inhabitant of highly urbanized regions, [2] but the necessity for population control also constitutes a risk for hunters to become infected by *Leptospira* sp. [8].

Considering these risks, the aim of this study was to estimate the prevalence of *Leptospira* infections in the population of wild boars in Poland.

Blood samples (n = 3621) were collected during the hunting seasons 2012–2014 in Poland. The samples originated from 314 counties from all the 16 provinces of Poland (Table 1). The sample size (population proportion) for each province was calculated according to Select Statistical Services program [9].

**Table 1:** Geographic distribution and seroprevalence for *Leptospira interrogans* in 3621 wild boars in 16 Polish provinces between 2012 and 2014

Provinces	No of samples	No of seropositive	% *L. interrogans* antibody positive (95 % CI)
DS	253	16	6.3 (3.9–10.0)
KP	268	38	14.2 (10.5–18.9)
LU	372	59	15.9 (12.5–19.9)
LB	218	23	10.6 (7.1–15.3)
LD	181	26	14.4 (10.0–20.2)
MP	187	9	4.8 (2.6–8.9)
MA	177	17	9.6 (6.1–14.8)
OP	182	10	5.5 (3.0–9.8)
PK	251	54	21.5 (16.9–27.0)
PD	163	19	11.7 (7.6–17.5)
PM	268	18	6.7 (4.3–10.4)
SL	187	29	15.5 (11.0–21.4)
SW	177	9	5.1 (2.7–9.4)
WM	198	10	5.1 (2.8–9.0)
WP	242	22	9.1 (6.1–13.4)
ZP	297	18	6.1 (3.9–9.4)
Total	3621	377	10.4 (9.5–11.4)

*DS* Lower Silesia, *KP* Kuyavian-Pomerania, *LB* Lubuskie, *LD* Łódzkie, *LU* Lubelskie, *MA* Masovia, *MP* Leser Poland, *OP* Opolskie, *PD* Podlaskie, *PK* Subcarpathia, *PM* Pomerania, *SL* Silesia, *SW* Świętokrzyskie, *WM* Warmia-Masuria, *WP* Greater Poland, *ZP* West Pomerania

Samples were collected during evisceration of wild boars shot during legal hunting; authority approval was therefore not required. Blood samples were taken for analysis from the large blood vessels in the neck area. Additionally, peritoneal fluid containing blood was collected. It was subjected to centrifugation at 5000*g* for 20 min to remove the cellular components of blood, tissue debris and bacterial contamination. The obtained supernatant was used for serological testing. Animals, which had been shot in the abdomen, were not included in the study. All samples were stored at −18 °C until analysis.

Serum samples were tested by microscopic agglutination test (MAT) using a range of ten *Leptospira* serovars representative of nine serogroups found in Europe: Icterohaemorrhagiae (strain RGA, representing serogroup Icterohaemorrhagiae), Grippotyphosa (strain Moskva V, serogroup Grippotyphosa), Sejroe (strain M84, serogroup Sejroe), Tarassovi (strain Perepelicyn, serogroup Tarassovi), Pomona (strain Pomona serogroup Pomona), Canicola (strain Hond Utrecht IV, serogroup Canicola), Bratislava (strain S/820834, serogroup Australis), Autumnalis (strain Akiyami, serogroup Autumnalis), Hardjo (strain Hardjoprajitno, serogroup Sejroe) and Ballum (strain MUS127, serogroup Ballum) [10, 11]. The reference strains were provided by the Veterinary Sciences Division, AFBI, OIE Leptospira Reference Laboratory, Belfast.

Each serovar was grown in 10 ml volumes of Ellinghausen-McCullough–Johnson-Harris (EMJH) medium, incubated at 28 °C for 6–10 days depending on the serovar. The concentration of bacteria was adjusted to 1–2 × 10^8^ cells/ml by cell count using a Helber counting chamber. The sera were initially screened for antibodies to the ten serovars at a final dilution of 1:100. When agglutination occurred, the relevant sera were end-point tested using doubling dilutions ranging from 1:100 to 1:25,600. The titre was defined as the highest dilution where ≥50 % of the antigen was agglutinated.

Calculation of Pearson’s and Spearman’s correlations and spatial analysis, STATISTICA (data analysis software system), version 10 (StatSoft, Inc.) and ArcGIS 10.1 SP1 for Desktop Standard (ESRI, Inc.) were used for data analyses. Wild boar demographics was derived from the Polish Hunting Association-PZL [12] and data were converted from province to county level.

**Fig. 1:**
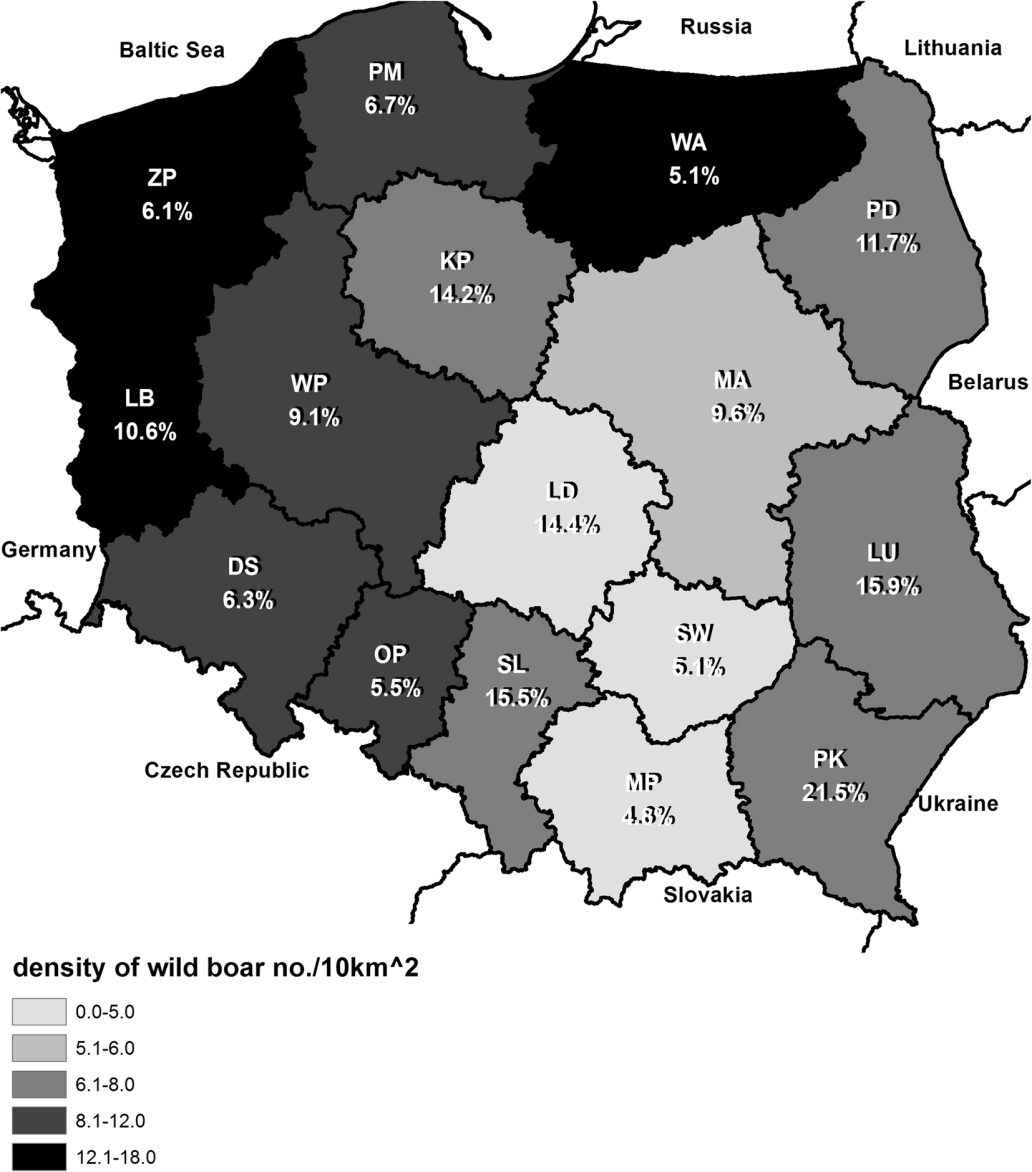
Geographic distribution of wild boars being seropositive for *Leptospira interrogans* in Poland. *DS* Lower Silesia, *KP* Kuyavian-Pomerania, *LB* Lubuskie, *LD* Łódzkie, *LU* Lubelskie, *MA* Masovia, *MP* Leser Poland, *OP* Opolskie, *PD* Podlaskie, *PK* Subcarpathia, *PM* Pomerania, *SL* Silesia, *SW* Świętokrzyskie, *WM* Warmia-Masuria, *WP* Greater Poland, *ZP* West Pomerania

Antibodies against *Leptospira* serovars were found in 377 samples (10.4 %). The highest prevalence was found in the province Subcarpathia (21.5 %), but other provinces also had wild boar populations with a high prevalence of *Leptospira* antibodies (Table 1; Fig. 1). Statistical analysis showed a statistically significant correlation between the seroprevalence and the density of wild boars (significance level alpha = 0.05) but the correlation was weak: Pearson’s correlation coefficient was equal to −0.20 (P = 0.010) and Spearman’s rank correlation coefficient confirmed the result of Pearson’s correlation and amounted to −0.20 (P = 0.013). The most common serovars were Hardjo, Pomona, Grippotyphosa and Bratislava (Table 2). Of the 377 *Leptospira* positive samples, 81 % had titers against one serovar, while 19 % had positive titers against two or more (≤7) serovars. These findings may be due to infections by more serovars or may reflect cross-reactions between strains of different serogroups [13]. The observed seroprevalence is at the same level as found in other European countries, especially Spain (12 %) [14] and Germany (18 %) [2]. In Germany, a high seroprevalence was found near the city of Berlin having a population of 3.5 million people [15]. Similarly, we found a high percentage of positive samples (15.5 %) in the Upper Silesian metropolitan area (population 2.7 million people) (Fig. 1) [15]. Data from Germany and Poland indicate that wild boars living close to city boarders with high human population density may be a threat to the inhabitants [2].

**Table 2:** Distribution of *Leptospira interrogans* antibody titers for 377 positive wild boars hunted during season 2012–2014 in Poland

	No of antibody-positive samples (%)						
Serovar	1:100	1:200	1:400	1:800	1:1600	1:3200	Total
Icterohaemorhagiae	8 (0.2)	3 (0.1)	6 (0.2)	0	0	0	17 (0.5)
Grippotyphosa	26 (0.7)	34 (0.9)	20 (0.6)	1 (0.03)	1 (0.03*)*	0	82 (2.3)
Sejroe	3 (0.1)	3 (0.1)	1 (0.03)	0	0	0	7 (0.2)
Tarassovi	32 (0.9)	9 (0.2)	2 (0.1)	2 (0.1)	0	0	45 (1.2)
Pomona	50 (1.4)	29 (0.8)	25 (0.7)	5 (0.1)	2 (0.1)	1 (0.03)	112 (3.1)
Canicola	14 (0.4)	1 (0.03)	0	0	0	0	15 (0.4)
Bratislava	29 (0.8)	20 (0.6)	9 (0,2)	5 (0.1)	1 (0.03)	0	64 (1.8)
Autumnalis	9 (0.2)	7 (0.2)	5 (0.1)	2 (0.1)	1 (0.03)	0	24 (0.7)
Hardjo	54 (1.5)	42 (1.2)	25 (0.7)	3 (0.1)	1 (0.03)	0	125 (3.5)

The seroprevalence of *Leptospira* in wild boars varies considerable across Europe from 65.4, 45.8 and 31.9 % in Portugal, [5], Slovenia [6] and Croatia [3], respectively to 2.6 % in Italy [16] and 3.1 % in Sweden [17]. The variation in seroprevalence across regions may be related to difference in populations of wild small mammals, which act as maintenance hosts for the various *Leptospira* serovars [18], although differences in study design and methods may also account for some of the differences. For example, recent studies carried out in northern Portugal demonstrated a high rate of titers against the new serovar Altodouro, which was isolated from *Mus musculus* in the region [19].

The seroprevalence of *Leptospira* infection in wild boars in Poland has been studied previously but that study was limited to the northern part of the country [20]. Unexpectedly, the results of our study indicated that the number of positive animals detected in this region was comparable to the findings of the previous study with an overall prevalence of infection in animals estimated at 25 %.

The population density of wild boars in many European countries including Poland has been continuously increasing for many years. During the last 30 years, this has resulted in a more than five-fold increase in the Polish wild boar population, initially reaching 46,000 animals in 1985 to more than 282,000 wild boars in 2015 [12]. An increasing population of wild boar results in more frequent contact between animals, which may lead to an increase in the spread of many infections.

Leptospirosis may be transmitted from wild boars to domestic pigs, although depending on pig production facilities. The serovars Pomona and Sejroe have been reported as the most common in domestic pigs in Poland [21, 22], but antibodies to these serovars were infrequent in our study (3.1 and 0.2 %, respectively) (Table 2). The study shows that wild boars in Poland are exposed to *Leptospira* sp., but that the seroprevalence varies across provinces. The serovars Hardjo and Pomona are the most common. The seroprevalence for *Leptospira* is particular high in the province of Subcarpathia. However, the epidemiology of leptospirosis in wild boars in Poland requires further studies, which should include isolation and typing of *Leptospira* strains.
